# New treatment options in elderly patients with Diffuse Large B-cell Lymphoma

**DOI:** 10.3389/fonc.2023.1214026

**Published:** 2023-07-03

**Authors:** Annalisa Arcari, Federica Cavallo, Benedetta Puccini, Daniele Vallisa

**Affiliations:** ^1^ Hematology and Bone Marrow Transplant Unit, Guglielmo da Saliceto Hospital, Piacenza, Italy; ^2^ Division of Hematology, Department of Molecular Biotechnologies and Health Sciences, University of Torino/Azienda Ospedaliera Universitaria (AOU) Città della Salute e della Scienza di Torino, Torino, Italy; ^3^ Hematology Department, University of Florence and Azienda Ospedaliera Universitaria (AOU) Careggi, Firenze, Italy

**Keywords:** elderly, lymphoma, geriatric assessment, chemotherapy, antibody

## Abstract

Most patients with Diffuse Large B-cell Lymphoma (DLBCL) are old (>65 years of age) and this population is expected to increase in the following years. A simplified geriatric assessment based on a careful evaluation of the fitness status and comorbidities is essential to choose the correct intensity of treatment. Fit older patients can benefit from a standard immunochemotherapy, while unfit/frail patients frequently need reduced doses or substitution of particular agents with less toxic ones. This review focuses on new therapies (e.g., polatuzumab vedotin, tafasitamab, bispecific antibodies) that have indicated promising results in relapsed/refractory patients, particularly in cases not eligible to transplant. Some of these new drugs have been tested as single agents or in combinations as first-line treatment, aiming to improve the outcome of the traditional chemotherapy. If preliminary efficacy and safety data are confirmed in future clinical trials, a chemo-free immunotherapic approach could become an alternative option to offer a curative treatment even in frail patients.

## Introduction

Diffuse Large B-cell Lymphoma (DLBCL) is the most frequent lymphoma subtype with a median age at diagnosis of 66 years (1). With the aging of the general population in Western countries, the number of old patients with lymphoma will continually increase, requiring specific considerations (2). Common issues in the treatment of geriatric patients are related to comorbidities and limited organ reserve (e.g., bone marrow, liver, and kidney) with a higher risk of toxicity. Clinicians should also consider issues related to impaired physical and/or cognitive functions that may compromise, especially in the absence of a care giver, the possibility to reach the hospital and to manage therapies at home. Besides factors regarding patients’ fitness status, an unfavorable biology of the disease may also contribute to an inferior outcome. DLBCLs in older patients are characterized by a higher prevalence of activated B-cell (ABC) subtypes and EBV-positive cases (3). The global prognosis of older DLBCL patients has certainly improved in the last few years thanks to immunochemotherapy combinations but is still poorer than in younger patients. Older patients are under-represented in clinical studies, particularly in clinical trials leading to marketing authorization of new cancer therapies (4).

## How to identify treatment goals for elderly DLBCL patients

Treating elderly patients with aggressive lymphoma poses the clinical dilemma of balancing a potential cure while minimizing toxicity. Age per se is not a contraindication to a full-dose curative treatment, but comorbid conditions and impaired functional status may often suggest a reduced dose and/or drugs substitution to improve tolerance. Elderly patients present a wide heterogeneity and traditional measures of performance status are not accurate enough to define treatment goals and to tailor treatment intensity. The ESMO guidelines recommend the application of a geriatric assessment to avoid the risk of undertreatment or overtreatment (5). The Fondazione Italiana Linfomi (FIL) has recently validated in a large prospective series of DLBCL patients older than 64 years –the Elderly Project- a simplified geriatric assessment (sGA) based on age (≥ or < 80 years), Cumulative Illness Rating Scale for Geriatrics (CIRS-G), activities of daily living (ADL), and instrumental activities of daily living (IADL) (6). This sGA is an objective, reproducible tool that can be easily managed by onco-hematologists (in less than 10 minutes) and permits to classify older patients as fit (55%), unfit (28%) or frail (18%), with significantly different outcomes.

## First-line treatment

### Fit patients

The aim of the first-line treatment in fit patients up to 80 years old should be curative, with a full dose anthracycline-based regimen. R-CHOP (rituximab, cyclophosphamide, doxorubicin, vincristine, prednisone) has been the standard initial therapy for more than 20 years (7). Attempts in improving the outcome of R-CHOP by adding new biological agents to immunochemotherapy, especially in non-GCB DLBCL, have failed to show a significant advantage (8). The PHOENIX trial did not demonstrate an improvement of event-free survival adding ibrutinib to R-CHOP in newly diagnosed non-GCB DLBCL; in patients > 60 years ibrutinib plus R CHOP was associated with an increase of toxicity, leading to a compromised R-CHOP administration and worse outcome (9). The ROBUST trial also failed to demonstrate an improvement with the combination of lenalidomide plus R-CHOP in untreated patients with ABC-type DLBCL (10).

The POLARIX study is the only randomised phase III trial in DLBCL which has shown a significant improvement so far of the progression-free survival (PFS) (11). In this trial, polatuzumab vedotin, an antibody-drug-conjugated targeting CD79b, replaced vincristine in the R-CHOP scheme. The new pola-R-CHP regimen showed a 2-year PFS of 76.7% compared to 70.2% of standard R-CHOP in intermediate-risk or high-risk DLBCL patients aged 18-80 years, with similar safety profiles. The overall survival (OS) rate at 2 years did not differ significantly (88.7% in the pola-R-CHP group versus 88.6% in the R-CHOP group). An exploratory subgroup analysis highlighted a stronger benefit in patients > 60 years, non-GCB types, double expressors, and high IPI (3–5). Considering the modest PFS advantage of pola-R-CHP and the equal OS, there are some concerns about a wide application of this regimen. Pola-R-CHP is a good option especially in specific patient subgroups, but probably R-CHOP will remain a standard arm in future clinical trials and a valid backbone for new combinations.

Most patients with high grade B-cell lymphoma (double or triple hit) are currently treated with the dose-adjusted R-EPOCH (rituximab, etoposide, prednisone, vincristine, cyclophosphamide, doxorubicin) regimen. However, this intensive therapy may not be suitable for patients older than 80 years and those < 80 years but with significant comorbidities (12, 13). For double/triple expressors cases there are no advantages using R-DA EPOCH in patients > 65 years (14).

### Unfit/frail patients

Unfit and frail patients mostly require an adapted therapy. The R-miniCHOP regimen can be considered the standard first-line treatment for DLBCL patients >80 years without severe comorbidities, with a 2-year PFS of 47% and a 2-year OS of 59% (15). The LYSA group has tried to improve the results of R-miniCHOP using ofatumumab instead of rituximab (16). The outcome in terms of 2-year OS (64.7%) was only slightly better than the previous study; nevertheless, this new protocol confirmed the importance of a systematic pre-phase with prednisone and vincristine before immunochemotherapy, which permits an improvement of the performance status and a reduction of treatment-related mortality during the first cycle (17). Based on the encouraging results of the POLARIX study, the Nordic Lymphoma Group is now conducting a randomized phase III trial comparing R-miniCHOP to R-miniCHP plus polatuzumab in patients ≥80 years or ≥75 years and frail (ClinicalTrials.gov Identifier: NCT04332822).

Many older patients have cardiac comorbidity and/or multiple cardiovascular risk factors (such as diabetes, hypertension, chronic renal disease) that are known to be associated with a higher risk of cardiotoxicity from anthracyclines (18, 19). In these cases, replacing conventional doxorubicin by the non-pegylated liposomal form may reduce the risk of cardiac events with a non-inferior efficacy (20–23).

For patients with a full contraindication to anthracyclines, the ESMO guidelines suggest the substitution of doxorubicin by gemcitabine or etoposide (24). The total omission of doxorubicin (such as in the R-CVP regimen) could be an option in older frail patients but the efficacy is generally low, and this option has a palliative aim (25). Tucci et al. recently reported that, within a palliative treatment, the use of rituximab may improve the outcome (2-yr OS with or without rituximab 42% vs. 22%; P=0.008) (26).

The safety and efficacy of rituximab plus bendamustine in indolent lymphoma has prompted its evaluation as first-line treatment in older frail patients with DLBCL. In a phase II trial of 49 DLBCL patients > 70 years with significant comorbidities and/or impaired fitness status, the overall response rate (ORR) was 62% (with 53% of complete remission rate, CRR), but the PFS was disappointing (38% at 2 years) (27). The combination rituximab plus lenalidomide (R2) has been tested in the phase II ReRi study in 68 newly diagnosed DLBCL patients not eligible for conventional cytotoxic therapy. The ORR was 41%, with PFS and OS at 12 months of 55% and 69%, respectively. Although this study did not confirm its initial end point, an activity was observed in a significant proportion of cases, warranting further exploration as backbone of new chemo-free combinations in elderly frail patients (28).

## Second-line treatment and beyond in relapsed/refractory patients

### Elderly patients eligible to transplant and/or CAR T cells

A limited number of elderly patients with relapsed/refractory DLBCL are eligible to the traditional standard approach based on salvage chemotherapy (in most cases with platinum-containing regimens) followed by high-dose therapy and autologous stem cell transplantation (ASCT) in case of chemosensitive disease, ideally in complete remission at PET/CT re-staging. The superior age limit in most studies was 60 or 65 years (29, 30). Only some small retrospective series, subgroup *post-hoc* analysis and data from international registries have described the outcome of older patients (31–33). In general, ASCT emerges from these studies as a feasible option in selected fit elderly patients up to 75 years of age.

Chimeric antigen receptor (CAR) T-cells have recently revolutionized the treatment landscape of aggressive lymphoma. Axicabtagene ciloleucel (axi-cel), tisagenlecleucel (tisa-cel) and lisocaptagene maraleucel (liso-cel) are currently FDA approved for the treatment of relapsed/refractory DLBCL patients after at least two prior lines of therapy. The pivotal trials ZUMA-1, JULIET and TRANSCEND NHL-001 showed high response rates (ORR 52-82%, CRR 40-54%) and durable complete remissions in about one third of infused patients (34–36). A sub-analysis of older patients enrolled in the ZUMA-1 trial highlighted a similar CAR T-cells *in vivo* expansion and an apparently higher efficacy in patients ≥65 years compared to patients <65 years (ORR 92% vs 81%, CRR 75% vs 53%, median PFS 13.2 months vs 5.6 months); the rate of grade ≥3 cytokine release syndrome (CRS), cytopenia and infections were similar, but older patients experienced more grade ≥3 neurotoxicity (44% vs 28%) (37). Recent real-world experiences confirmed that the outcome of CAR T-cells therapy is comparable between older and younger patients, indicating that age itself should not preclude CAR T-cells administration; a careful evaluation of comorbidities, a reliable caregiver and a longer rehabilitation therapy may be essential to improve the long-term outcome (38–40). Transplant-ineligible but CAR T-eligible elderly patients could become a real and relevant population in a near future (41).

Based on the favorable results of the ZUMA-7 trial, comparing CAR T-cells to the standard of care (two or three cycles of salvage chemotherapy followed by ASCT), axi-cel has been recently approved as second-line therapy in adult patients with large B-cell lymphoma who are refractory to first-line chemoimmunotherapy or relapsed within 12 months (42). In this trial, patients aged ≥65 years were 51 (28%) in the axi-cel arm and 58 (32%) in the standard arm. In the TRANSFORM trial, liso-cel proved its superiority versus standard of care as second-line therapy in refractory or early relapsed DLBCL patients, while the BELINDA trial did not reach the same end point with tisa-cel (43, 44).

Despite a significant efficacy, many issues can limit the widespread application of CAR T-cells in clinical practice, particularly in older patients: the necessity of specialized centers that may be far from the patient’s residence, the long turnaround time from the leukapheresis to product release, and the cost of the entire treatment.

### Elderly patients not eligible to transplant or CAR T-cells

Elderly patients with relapsed/refractory DLBCL not eligible to transplant have a dismal prognosis with conventional second-line treatments such as rituximab-gemcitabine-oxaliplatin (R-GEMOX), bendamustine-rituximab (BR), pixantrone, and lenalidomide, with an ORR of 35-50% and a median PFS of 4-8.8 months (45–48).

In recent years, novel agents have emerged as potentially more effective therapies in this difficult-to treat setting (**Table 1**). Polatuzumab vedotin is a new antibody-drug conjugated, that delivers monomethyl auristatin E (MMAE), a microtubule inhibitor, to B-cells, targeting the CD79b antigen (49). A phase II study randomly assigned 80 patients with relapsed/refractory transplant-ineligible DLBCL to the combination polatuzumab-BR versus BR alone (50). The median number of prior lines of therapy was 2 (range 1-7) and most patients (75-85%) were refractory to the last treatment. Polatuzumab-BR showed a significantly higher CR rate (40.0% *vs* 17.5%), longer PFS (9.5 *vs* 3.7 months; p < 0.001) and OS (12.4 *v* 4.7 months; p = 0.002) compared to BR alone. Pola-BR patients had higher rates of hematological toxicities but similar grade 3-4 infections. Peripheral neuropathy, typically associated with MMAE, was grade 1-2 in all cases and resolved in most patients. Updated results from the randomized arms with a median follow up of 48 months and results of an extension cohort of 106 additional patients that received pola-BR confirmed a significant survival benefit; no new safety signals were identified (51).

**Table 1:** Clinical trials in relapsed/refractory DLBCL patients not eligible to transplant.

	Pola-BR (n=152 pts)	Tafasitamab-lenalidomide (n=81 pts)	Loncastuximab (n= 145 pts)
Study design	phase 1b/2, random + extension cohort, ≥2 L	phase 2, non random, ≥2 L	Phase 2, non random, ≥ 3 L
**ORR (IRC)**	41,5%	57,5%	48,3%
**CR**	38,7%	40%	24%
**PR**	2,8%	17,5%	24%
**SD**	3,8%	16,3%	15%
**PD**	17,9%	16,3%	21%
**mPFS (months)**	6,6	11,6	4,9
**mDOR (months)**	9,5	43,9	10,3
**mOS (months)**	12,5	33,5	9,9
**Adverse events (grade ≥3)**	neutropenia (32,5%), infections (21,9%), thrombocytopenia (20,5%), anemia (12,6%)	neutropenia (49,4%), thrombocytopenia (17,3%), febrile neutropenia (12,3%)	neutropenia (26%), thrombocytopenia (18%), increased gamma-glutamyl transferase (17%)

Sehn et al. Blood adv 2021; Duell et al. Haematologica 2021; Caimi et al. Lancet Oncol 2021.

DLBCL, diffuse large B-cell lymphoma; Pola-BR, polatuzumab-bendamustine-rituximab; ORR, overall response rate; IRC, independent review committee; CR, complete remission; PR, partial remission; SD, stable disease; PD, progressive disease; mPFS, median Progression Free Survival; mDOR, median Duration of Response; mOS, median Overall Survival; random, randomized trial; L, prior lines of therapy.

A second possible salvage option for DLBCL patients not eligible to transplant or CAR-T cells is the combination tafasitamab-lenalidomide. Tafasitamab is a new anti-CD19 antibody with an enhanced Fc-portion. Although tafasitamab and lenalidomide have a limited single-agent activity, *in vitro* and *in vivo* studies showed a synergistic effect with limited toxicity (52). The phase II, single-arm, L-MIND study enrolled 81 patients with DLBCL relapsed after 1-3 prior systemic regimens; primary refractory cases were only a minority of the cohort (53). Tafasitamab was administered in combination with lenalidomide for 12 cycles, followed by tafasitamab monotherapy until progression or toxicity. An updated analysis with ≥35 months of follow up showed an ORR of 57.5%, including CR in 40% of cases; the median OS was 33.5 months and the median PFS was 11.6 months. Therefore, tafasitamab-lenalidomide seems to permit a long duration of response with a well-tolerated immuno-modulatory combination (54).

The CD-19 antigen is also the target of loncastuximab tesirine, a new antibody-drug conjugated that delivers pyrrolobenzodiazepine (PBD) dimers after binding to B-cell surface and entering the cell (55). The phase II LOTIS-2 study enrolled 145 DLBCL patients relapsed or refractory after at least 2 prior lines of treatment; 20% of patients were primary refractory, 20% had transformed lymphoma and 10% double/triple hit lymphoma. The ORR in this heavily pre-treated and high-risk cohort was 48.3%, with CR in 24.1% of cases and a median duration of response of 10.3 months. The safety profile was acceptable with neutropenia, thrombocytopenia, increased gamma-glutamyl transferase and pleural effusions as most relevant adverse events (56).

The choice between these different options could be quite difficult in clinical practice. Real-world data described outcomes not as good as that seen in clinical trials, probably due to less selected patients (57, 58). In the absence of randomized trials, a comparison between agents only derives from retrospective, matched cohorts and results should be interpreted with caution. Main factors to be considered for treatment decision are: the aim of therapy (complete remission, duration of response, quality of life), patients’ characteristics (age, fitness, comorbidities), logistic and social aspects (presence of caregiver, distance from the hospital), disease characteristics (prior lines of treatment, refractoriness).

The B-cell lymphoma treatment landscape has recently been broadened by bispecific anti CD20xCD3 antibodies that can engage and redirect patients’ T-cells to eliminate malignant B-cells (59–62; **Table 2**). The main advantage is an off-the-shelf rapid availability and a toxicity similar but generally inferior to CAR T-cells. Glofitamab, a bispecific antibody characterized by a novel 2:1 CD20-CD3 binding configuration, has shown a high response rate (ORR 52%, CRR 39%) in 154 DLBCL patients (median age 66 years) relapsed or refractory after at least two prior lines of treatment, of which 52 had already received CAR T-cells therapy; complete remissions were ongoing at 12 months in 78% of cases. The most frequent adverse event, common to this class of agents, was CRS (all grades 63% of patients, grade ≥3 4%) (59). Epcoritamab is of particular interest in elderly patients, thanks to its subcutaneous administration. In the dose-expansion cohort of a phase I/II study, 157 DLBCL patients (median age 64 years) were treated, showing an ORR of 63%, a CRR of 39%, and a median duration of response of 12 months with continuous therapy. The CRS was frequent, but of grade 1-2 in most cases (97%) (60).

**Table 2:** Phase I/II trials with bispecific antibodies in relapsed/refractory DLBCL patients.

Drug	Phase	N. of patients	Response Rates	CRS gr ≥3	ICANS ≥3
**Glofitamab**	I/II	154	ORR 52% CRR 39%	4%	3%
**Mosunetuzumab**	I/Ib	129	ORR 35% CRR 19%	1%	1%
**Epcoritamab**	I/II	157	ORR 63% CRR 39%	2.5%	0.6%
**Odronextamab**	II	121	ORR 53% CRR 37%	0%*	0%*

Dickinson et al. NEJM 2022; Thieblemont et al. J Clin Oncol 2022; Budde et al. J Clin Oncol 2022; Kim et al. 64th American Society of Hematology (ASH) Annual Meeting and Exposition

DLBCL, diffuse large B-cell lymphoma; ORR, overall response rate; CRR, complete remission rate; CRS, cytokine release syndrome; ICANS, immune effector cell-associated neurotoxicity syndrome.

*after revision of the original step-up dosing program.

## Future perspectives

As mentioned previously, many efforts to improve the standard R-CHOP regimen by adding novel targeted agents (the so-called R-CHOP plus X trials) have failed to demonstrate a better OS. Future trials will attempt to achieve better results combining R-CHOP with novel bruton tyrosin kinase inhibitor characterized by a more favorable profile, such as zanubrutinib (ClinicalTrials.gov Identifier: NCT05189197). The front-MIND phase III trial (ClinicalTrials.gov Identifier: NCT04824092) will compare efficacy and safety of tafasitamab-lenalidomide plus R-CHOP versus R-CHOP alone in newly diagnosed DLBCL patients aged 18-80 years, with high-intermediate or high-risk disease.

A different strategy, proposed by investigators from the MD Anderson Cancer Center, is based on an initial phase with biological agents alone (RLI: rituximab, lenalidomide, and ibrutinib) administered for two cycles to patients with non-GCB DLBCL, followed by the addition of conventional chemotherapy (either R-CHOP or R-EPOCH). With the limits of a small number of patients (60 in total, of which 28% ≥70 years), this “Smart Start study” paths the way for a targeted therapy before chemotherapy, showing an impressive response rate after RLI alone (ORR 86%, CRR 36%). The entire program resulted in an ORR of 100% and a 2-year PFS of 91% (63).

For unfit/frail elderly patients not eligible to standard chemotherapy, an emerging approach is a “chemo-free” treatment, based on new antibodies and small molecules. A phase I/II study explored the use of the bispecific antibody mosunetuzumab as first-line treatment in DLBCL patients >80 years or >60 years but with comorbidities precluding full-dose chemoimmunotherapy. ORR and CRR were 56% and 43%, respectively, in 54 patients with a median age of 83 years; no grade ≥3 CRS and no neurotoxicity were reported (64). An incoming phase II trial will assess epcoritamab alone or in combination with lenalidomide as first-line treatment in elderly DLBCL patients who are considered anthracycline ineligible (ClinicalTrials.gov Identifier: NCT05660967).

## Conclusions

The management of elderly patients with aggressive lymphoma continues to be a challenge, but a new era has been opened. Objective parameters that define the fitness status of the patient are fundamental to establish the correct treatment intensity and should be included in future clinical trials. A quality-of-life assessment and patient-reported outcomes should also be considered as crucial end points. New drugs, with immunological mechanisms of action, could help improve the outcome of patients relapsed or refractory after standard chemotherapy or those not eligible to standard chemotherapy because of comorbidities.

## Author contributions

AA was responsible for the scientific concept of the review. AA and FC wrote the manuscript. All authors (AA, FC, BP, DV) contributed to the literature review and critical revision of the manuscript, and approved the submitted version.
